# Clarification of the Identity of the Tea Green Leafhopper Based on Morphological Comparison between Chinese and Japanese Specimens

**DOI:** 10.1371/journal.pone.0139202

**Published:** 2015-09-30

**Authors:** Daozheng Qin, Li Zhang, Qiang Xiao, Christopher Dietrich, Masaya Matsumura

**Affiliations:** 1 Key Laboratory of Plant Protection Resources and Pest Management of the Ministry of Education, Entomological Museum, Northwest A&F University, Yangling, Shaanxi, China; 2 Tea Research Institute, Chinese Academy of Agricultural Sciences, Hangzhou, Zhejiang, China; 3 Illinois Natural History Survey, Prairie Research Institute, University of Illinois, Champaign, Illinois, United States of America; 4 Kyushu Okinawa Agricultural Research Center, National Agriculture and Food Research Organization, Koshi, Kumamoto, Japan; Institute of Zoology, CHINA

## Abstract

Tea green leafhopper is one of the most dominant pests in major tea production regions of East Asia. This species has been variously identified as *Empoasca vitis* (Goëthe), *Jacobiasca formosana* (Paoli) and *Empoasca onukii* Matsuda in Mainland China, Taiwan and Japan, respectively. Recent study of DNA sequence data suggested that treatment of this pest as different species in these three adjacent regions is incorrect and that they were a single species; but the correct scientific name for the species has remained unclear. Consistent with the prior molecular evidence, morphological study shows that the male genital characters of Chinese specimens are the same as those of specimens from Japan, so the correct scientific name of tea green leafhopper in China is *Empoasca* (*Matsumurasca*) *onukii* Matsuda.

## Introduction

As a fundamental science of identifying, describing, and classifying living organisms, taxonomy is one of the most important sub-disciplines of the life sciences [1]. Without accurate taxonomic identification, research carried out in academic and applied branches of life sciences is effectively unreliable [2]. Misidentifications of insect pests applied by Chinese scientists in recent years have resulted in confusion over the true identities of such species. One example is tea green leafhopper, the most dominant pest in Chinese tea plantations.

Tea green leafhopper belongs to the cosmopolitan genus *Empoasca* Walsh, 1862, a rather diverse, complex group in the tribe Empoascini of the family Cicadellidae currently comprising more than 1000 described species. The species-level classification of the genus is in need of comprehensive revision because of the small size of the genus, the necessity for clearing and examining male genitalia and other abdominal structures, and inability to identify females using morphological characters. Furthermore, species descriptions are scattered over dozens of publications, no inclusive revision of *Empoasca* has been attempted, and no comprehensive keys to species of *Empoasca* have been published. Body coloration may be considered at best a supporting character for species recognition [3,4].

In East Asia, the economic losses caused by tea green leafhopper are considerable with an average of 15–50% annual yield loss in Mainland China and Taiwan. In addition, tea quality may be severely reduced by feeding injury to leaves caused by the leafhopper [5,6]. Although tea green leafhopper has been known as one of the most serious pests in Mainland China since the 1950s [7], the taxonomic identity of the species has long been uncertain, with two scientific names, *Empoasca flavescens* (Fabricius) and *Empoasca pirisuga* (Matsumura) variously adopted by applied entomologists until the end of 1980s [8,9]. Kuoh & Zhang (1988) attempted to clarify the identity of the species after checking specimens from 11 tea producing provinces (regions) in south China, identifying the main pest in Chinese tea plantations as *Empoasca vitis* (Goëthe) [10]. This identification has been widely accepted by more recent researchers [11–15]. Subsequently, tea researchers identified the tea green leafhopper in Taiwan as *Jacobiasca formosana* (Paoli) [16]. In Japan, it was identified as *Empoasca onukii* Matsuda [17, 18].

Despite the previous studies reporting that the dominant tea pest in Shaanxi Province (northwest China) was *Empoasca vitis* [10,19], Qin *et al*. (2014) identified the leafhopper species in this area as *Empoasca* (*Matsumurasca*) *onukii* Matsuda, the same species as reported injurious to tea in Japan, based on examination of male genital structures. They provided a brief morphological description of this species and suggested that the tea green leafhopper elsewhere in Mainland China could be *Empoasca* (*Matsumurasca*) *onukii* rather than *Empoasca vitis* [20]. Shortly thereafter, Shi *et al*. (2014) re-examined tea green leafhopper from Fujian Province (southeast China) and agreed with the identification of Qin *et al*. (2014) [21].

Despite the findings of Qin *et al*. (2014) and Shi *et al*. (2014), the name *Empoasca vitis* is still widely applied to tea green leafhoppers by many Chinese scientists in current projects and papers [22–24]. New evidence from molecular data, has suggested that there was a single species of tea green leafhopper in Mainland China, Taiwan and Japan, rather than the three species recognized by researchers working in these adjacent regions [25]. The molecular research was conducted “for lack of recognized morphological characters.” The current study aimed to clarify the taxonomic identity of the tea green leafhopper and morphological characters distinguishing from other species of *Empoasca*.

Tea trees are planted in 21 provinces and regions in China [26] and tea is also one of the major crop plants in Japan [27]. Considering the economic importance in agriculture and the increasing number of papers concerned with this pest (with incorrect name) in recent years, it is important to clarify the features of this pest that facilitate accurate identification. In the present paper, we identify this species based on a large number of specimens collected from Mainland China, Taiwan and Japan.

## Materials and Methods

### Ethics Statement

No specific permits were required for this study. Tea green leafhopper is an agricultural pest, not an endangered or protected species. All samples were collected in open tea plantations and not from any national parks or protected areas.

### Specimen Collection

Tea green leafhopper specimens in China (including Taiwan) were collected in 18 provinces (regions) during the seasons of tea green leafhopper occurrence from 2011 to 2014. The specimens from Japan were collected from August to October in 2014 in Kagoshima Prefectural Institute for Agricultural Development, Kyushu (Table 1).

**Table 1:** Collecting information of tea green leafhoppers from Mainland China, Taiwan and Japan.

Collecting locality		Latitude(N)/ Longitude(E)	Collecting date (M/Y)	Number of male individuals
Shandong	Qingdao	36°16.2'/120°38.23'	8/2013	194
	Jiaonan	35°39.53'/119°34.18'	8/2013	49
	Rizhao	35°17.02'/119°16.00'	7/2013	124
	Taian	36°14.00'/117°11.95'	8/2013	234
Henan	Xinyang	31°45.90'/114°40.03'	7/2013	230
	Xinxian	31°42.87'/114°59.75'	7/2013	156
Shaanxi	Hanzhong	32°57.00'/107°40.11'	7/2013	189
	Ankang	32°30.18'/108°27.17'	8/2011	12
Jiangsu	Wuxi	31°29.47'/120°18.70'	6/2012	6
	Nanjing	32°0.30'/118°46.80'	9/2014	30
Zhejing	Hangzhou	30°13.20'/120°05.02'	9/2014	257
	Jinhua	28°53.82'/119°49.02'	9/2014	106
Anhui	Xuancheng	30°47.15'/119°03.23'	12/2004	42
	Huangshan	29°51.13'/117°42.95'	9/2014	157
Jiangxi	Yichun	28°39.27'/114°33.62'	7/2014	26
	Nanchang	28°48.07'/115°43.27'	7/2014	28
Hubei	Enshi	30°28.60'/109°16.30'	7/2013	19
	Yichang	30°57.85'/110°59.77'	7/2014	82
Hunan	Changsha	28°31.62'/113°22.52'	7/2014	52
	Changde	28°38.60'/111°09.67'	7/2014	116
Chongqing	Yongchuan	29°23.38'/105°54.25'	5/2014	126
	Rongchang	29°24.07'/105°29.78'	5/2014	109
Guizhou	Guiyang	26°38.01'/106°37.02'	8/2010	7
	Zunyi	27°45.65'/107°29.33'	7/2014	56
Sichuan	Leshan	29°46.50'/103°40.50'	9/2013	69
	Chengdu	30°31.35'/103°25.18'	8/2013	156
Yunnan	Chuxiong	24°32.62'/101°49.78'	7/2014	107
	Baoshan	25°07.03'/98°32.42'	9/2011	6
Fujian	Fuzhou	26°05.08'/119°14.37'	6/2014	62
	Wuyishan	27°44.82'/117°40.67'	8/2011	3
Guangdong	Yingde	24°11.12'/113°24.25'	11/2012	28
Guangxi	Guilin	25°22.50'/110°55.80'	6/2012	10
Hainan	Danzhou	19°3.01'/109°29.02'	5/2012	10
Taiwan	Hsinchu	24°42.67'/121°3.63'	8/2014	10
Japan	Kagoshima	31°36.52’/130°44.76’	8-10/2014	54

To keep sample contamination to a minimum (i.e., reduce the number of non-tea-feeding species of the polytypic genus *Empoasca* captured), specimens were collected from tea plants by sweep net rather than by light trapping; all the specimens were carefully collected near the central areas in tea plantations to avoid capturing insects feeding on weeds and other non-tea plants. All these specimens were preserved in absolute alcohol and deposited in the Entomological Museum, Northwest A&F University, Yangling, China (NWAU). Additional fresh adult specimens collected in Hangzhou, Zhejiang Province (southeast China) on March 25, 2015 were photographed to show the green color of live specimens, which fades to yellow after preservation.

To study the morphological characters, ten male adults were randomly selected from each province (region) in China and Japan, all these specimens were observed and dissected under an OLYMPUS SZX-10 Stereoscopic Zoom Microscope. Male genitalia were prepared as described by Oman (1949) [28]. Photographs of adult and wings were taken with an OLYMPUS PM-10 AD microscope, and male genitalia were taken by using an automontage QIMAGING Retiga 4000R digital camera (CCD) stereozoom microscope. Images were produced using the software Auto-Montage Pro. All pictures were edited using Adobe Photoshop CS7.0 (Adobe Systems). Body measurements are from apex of vertex to tip of forewing.

Morphological terminology used in this work follow Zhang (1990) [29] with the following exceptions: wing venation follows Dworakowska (1993) [30], groups of setae on the subgenital plate follow Southern (1982) [4], leg chaetotaxy follows Rakitov (1998) [31].

## Results

### Tea Green Leafhopper in Japan

**Fig 1:**
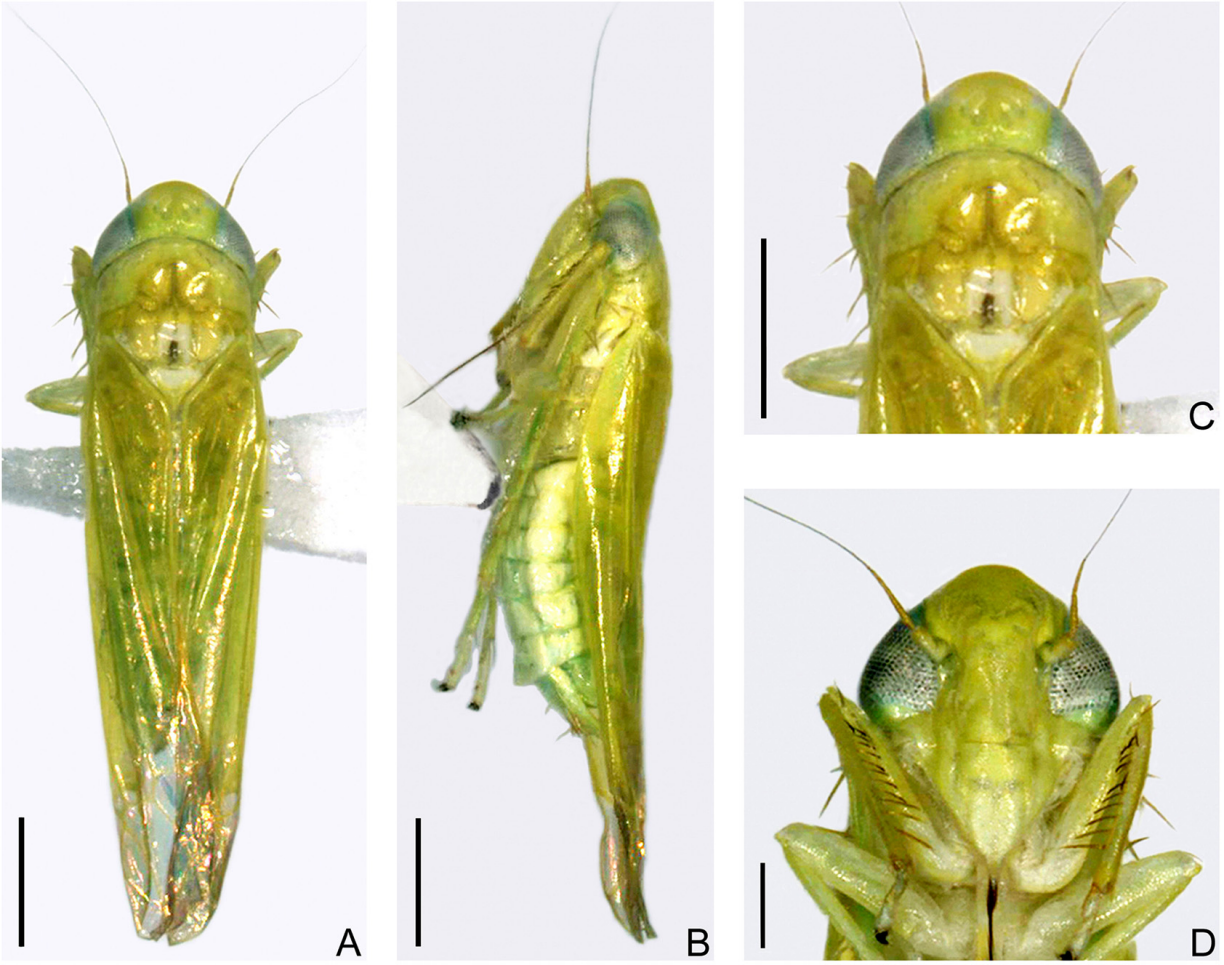
Adult of *E*. (*M*.) *onukii* Matsuda (alcohol preserved specimens from Kagoshima, Japan). (**A**) Male adult, dorsal view. (**B**) Female adult, left lateral view. (**C**) Head and thorax, dorsal view. (**D**) Face. Scale bars: (**A**)–(**C**) = 0.5 mm; (**D**) = 0.2 mm.

**Fig 2:**
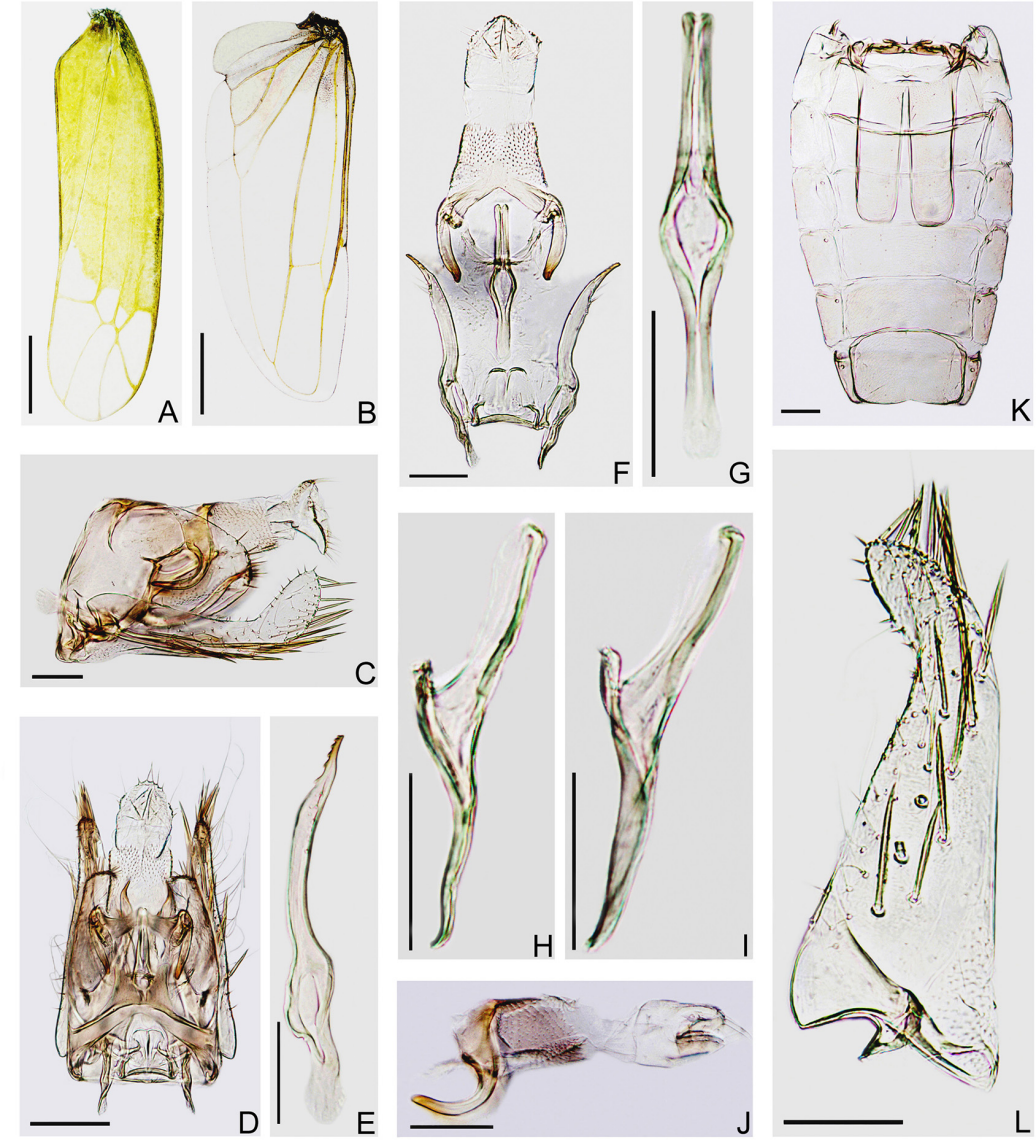
Wings and male genitalia of *E*. (*M*.) *onukii* (specimens from Kagoshima, Japan). (**A**) Forewing. (**B**) Hind wing. (**C**) Male genitalia, left lateral view. (**D**) Male genitalia, dorsal view. (**E**) Paramere. (**F**) Anal tube, anal styli, aedeagus, connective and parameres, dorsal view. (**G**) Aedeagus, ventral view. (**H, I**) Aedeagus, left lateral view. (**J**) Anal tube and anal styli, left lateral view. (**K**) Abdominal apodemes. (**L**) Subgenital plate. Scale bars: (**A, B**) = 0.5 mm; (**C**)–(**L**) = 0.1 mm.

The diagnosis of the tea green leafhopper from Japan was unclear before 1970s because the morphological description and illustrations of Matsuda (1952) were insufficient to show diagnostic features of the male genitalia [17]. This species became distinguishable after the work of Dworakowska (1971), who checked the male genital apparatus of the holotype and re-illustrated this species [32]. The Japanese leafhopper specimens in this study are identifiable as *Empoasca* (*Matsumurasca*) *onukii* Matsuda based on the diagnostic features of male genitalia (aedeagus, paramere, anal tube appendage and abdominal apodemes) as shown by Dworakowska (1971) [32] (Figs 1 and 2).

### Identity of Tea Green Leafhopper in Mainland China and Taiwan

At least 180 leafhopper specimens collected from Chinese tea plantations in 18 provinces (regions) were dissected and identified, with attention paid to male genital characters, and any morphological variation evaluated. The results showed that all these structures were in agreement with those of the leafhopper specimens from Japan. All *Empoasca* specimens from Chinese tea plantations were *E*. *onukii*; no other species was found in our samples. The correct scientific name of tea green leafhopper in China is, therefore, *Empoasca* (*Matsumurasca*) *onukii* Matsuda.

The collecting sites of Chinese leafhopper specimens in this study covered all the provinces (regions) that Kuoh & Zhang (1988) had studied. Beside this, Qin *et al*. (2014) evaluated the re-illustrations of Kuoh & Zhang (1988) which confirmed that the main structures (aedeagus and anal tube appendage) corresponded more with those of *E*. (*M*.) *onukii* than with *Empoasca vitis* [20]. Moreover, specimens from Japan were available to help identify the Chinese species. For the above reasons, the present study shows that Kuoh & Zhang (1988) misidentified this pest.

Liu *et al*. (2011) redrew the male genitalia in a revisionary work of the subgenus *Empoasca* (*Matsumurasca* Anufriev) in China [33] and Qin *et al*. (2014) presented a brief morphological re-description of this pest based on the specimens from Shaanxi Province [20]. We present a more detailed re-description and re-illustration of this species below, including some important features not mentioned in previous descriptions.

### Species Description

*Empoasca* (*Matsumurasca*) *onukii* Matsuda

*Empoasca onukii* Matsuda, 1952: 20 [17].

*Chlorita onukii*, Ishihara, 1953: 30 [34].

*Empoasca* (*Chlorita*) *onukii*, Metcalf, 1968: 573 [35].

*Empoasca* (*Matsumurasca*) *onukii*, Dworakowska, 1971: 505 [32]; Dworakowska, 1982: 52 [36]; Qin & Zhang, 2008: 24 [37]; Liu *et al*., 2011: 31 [33]; Qin *et al*., 2014: 125 [20].

#### Length

Total length (from apex of vertex to the tip of forewing) male 2.25–3.14 mm (N = 180); female 2.25–3.46 mm (N = 180); body length (from apex of vertex to the tip of male or female genitalia); male 2.35–2.83 mm (N = 180), female 2.52–3.27 mm (N = 180); forewing length male 2.13–2.64 mm (N = 180), female 2.32–3.05 mm (N = 180).

#### Color

**Fig 3:**
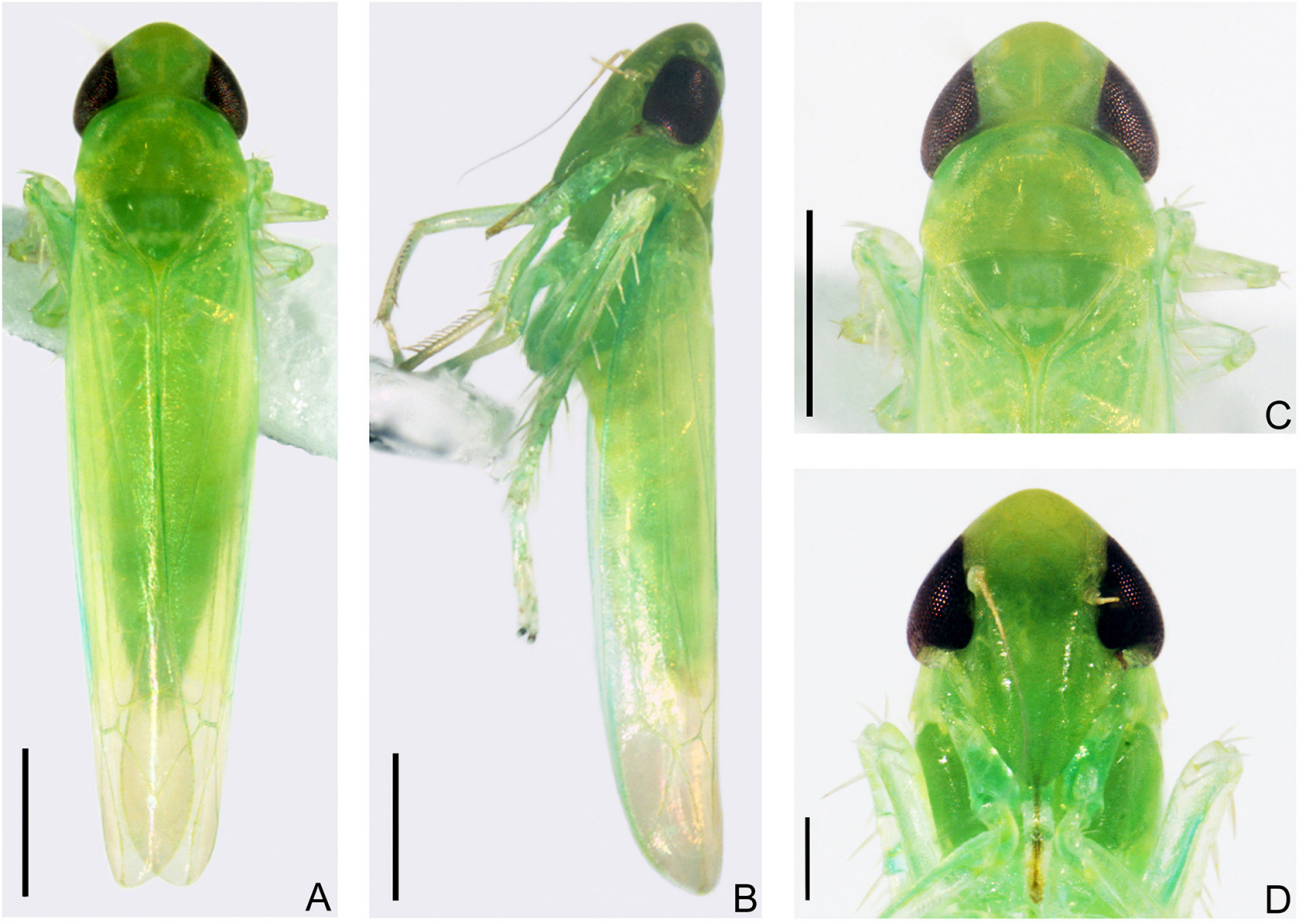
Adult of *E*. (*M*.) *onukii* (fresh specimens from Hangzhou, China). (**A**) Male adult, dorsal view. (**B**) Male adult, left lateral view. (**C**) Head and thorax, dorsal view. (**D**) Face. Scale bars: (**A**)–(**C**) = 0.5 mm; (**D**) = 0.2 mm.

Male. Predominant color of fresh specimens pale green to yellow, with or without few symmetrical creamy markings and greenish hypodermal patches on crown (Fig 3A–3D). Eyes grayish brown to black (Fig 3A–3D). Ocelli circled by narrow greyish patch, creamy patch mesocaudad distinct or absent (Fig 3A and 3C). Pronotum with or without lighter and irregular creamy patches along anterior margin and arcuate area behind eyes (Fig 3A and 3C). Centre of scutellum antero-mesally, caudad of scutoscutellar sulcus and at each side of lateral margins usually with greyish or creamy patches (Fig 3A and 3C). Face concolorous or yellow at base and indigo at apex of anteclypeus (Fig 3D). Forewing semitransparent in basal 2/3, apical third and hind wing hyaline (Fig 3A and 3B). Legs yellow but usually bearing indigo pattern in some segments (Fig 3B). Female usually with the same color as male, ovipositor grayish to brown. Old and alcohol-preserved specimens generally yellow, with color and markings usually faded.

#### Structure

Head including eyes slightly narrower to nearly same as maximum width of pronotum in dorsal aspect (Fig 3A and 3C). Crown rounded anteriorly, longer submedially than next to eye, shorter than width between eyes, anterior and posterior margins subparallel, width between eyes greater than eye width; coronal suture distinct, not attaining anterior margin of crown (Fig 3A and 3C). Ocelli on margin and close to eyes (Fig 3A and 3C). Face broad, slightly narrower than median length, postclypeus slightly convex in profile, lateral frontal sutures extended beyond antennal pits but not to midline (Fig 3B and 3D). Rostrum at most reaching hind coxae (Fig 3D). Pronotum large, distinctly longer than crown in midline (Fig 3A and 3C). Front femur with dorsoapical pair of macrosetae, AM1 distinctly enlarged, intercalary row with 1 large basal seta and ~8 smaller setae more distad. Middle femur with 1 dorsoapical macroseta. Hind femur macrosetae 2+1+1, tibia row AV with 5 macrosetae near apex.

#### Wings

**Fig 4:**
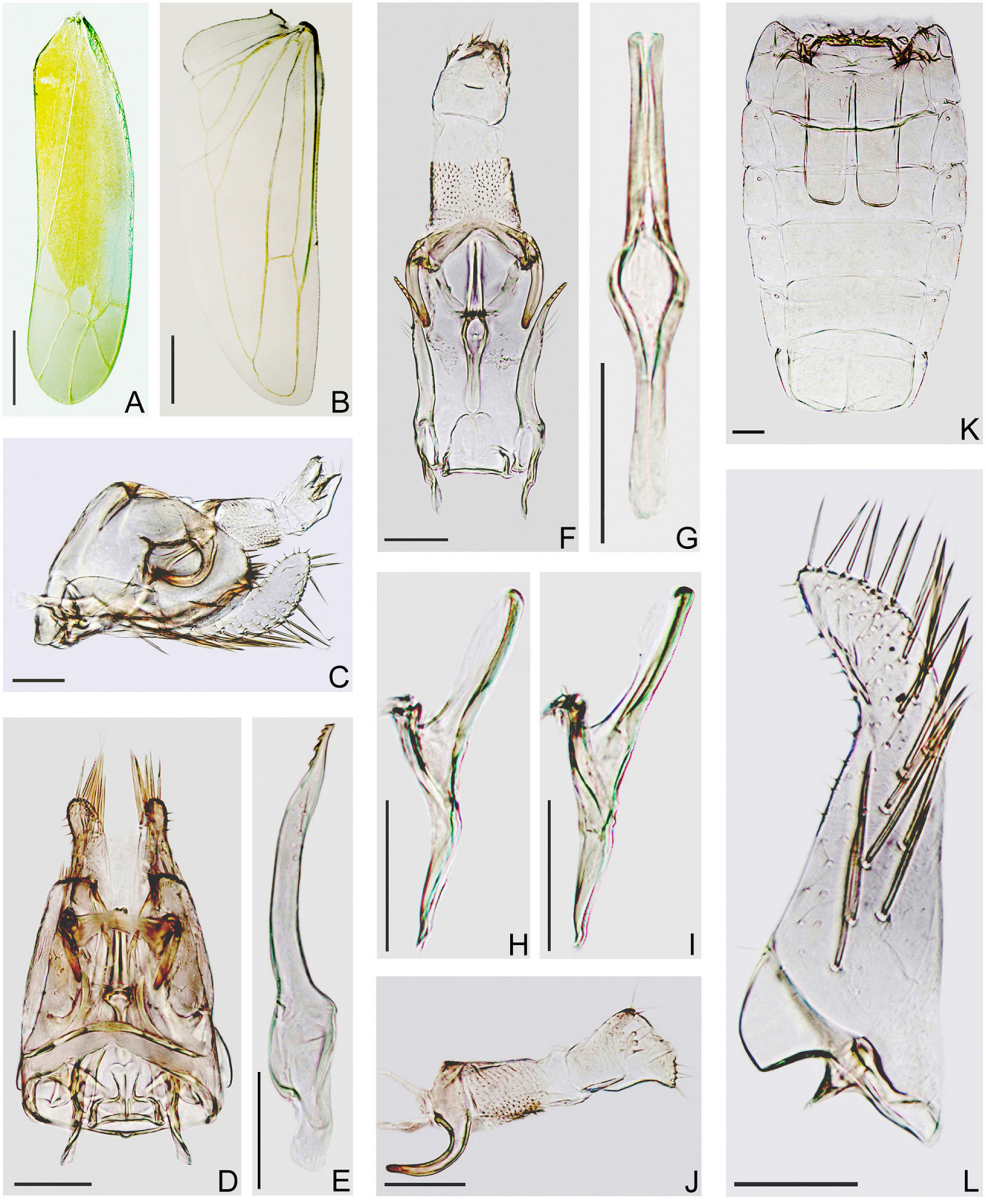
Wings and male genitalia of *E*. (*M*.) *onukii* (specimens from China). (**A**) Forewing. (**B**) Hind wing. (**C**) Male genitalia, left lateral view. (**D**) Male genitalia, dorsal view. (**E**) Paramere. (**F**) Anal tube, anal styli, aedeagus, connective and parameres, dorsal view. (**G**) Aedeagus, ventral view. (**H, I**) Aedeagus, left lateral view. (**J**) Anal tube and anal styli, left lateral view. (**K**) Abdominal apodemes. (**L**) Subgenital plate. Scale bars: (**A, B**) = 0.5 mm; (**C**)–(**L**) = 0.1 mm.

Forewing narrow, rounded apically, apical cells less than one-third of total length, 2nd apical cell with margins subparallel at bases but apparently broadened apically; 3rd apical cell petiolate or triangulate, 4th apical cell shortest, veins RP, MP’ arise from r cell and MP”+CuA’ from m cell, c and r cells nearly equal in width, both narrower than m and cua cells (Fig 4A). Hind wing with CuA not branched (Fig 4B).

#### Male genitalia

**Fig 5:**
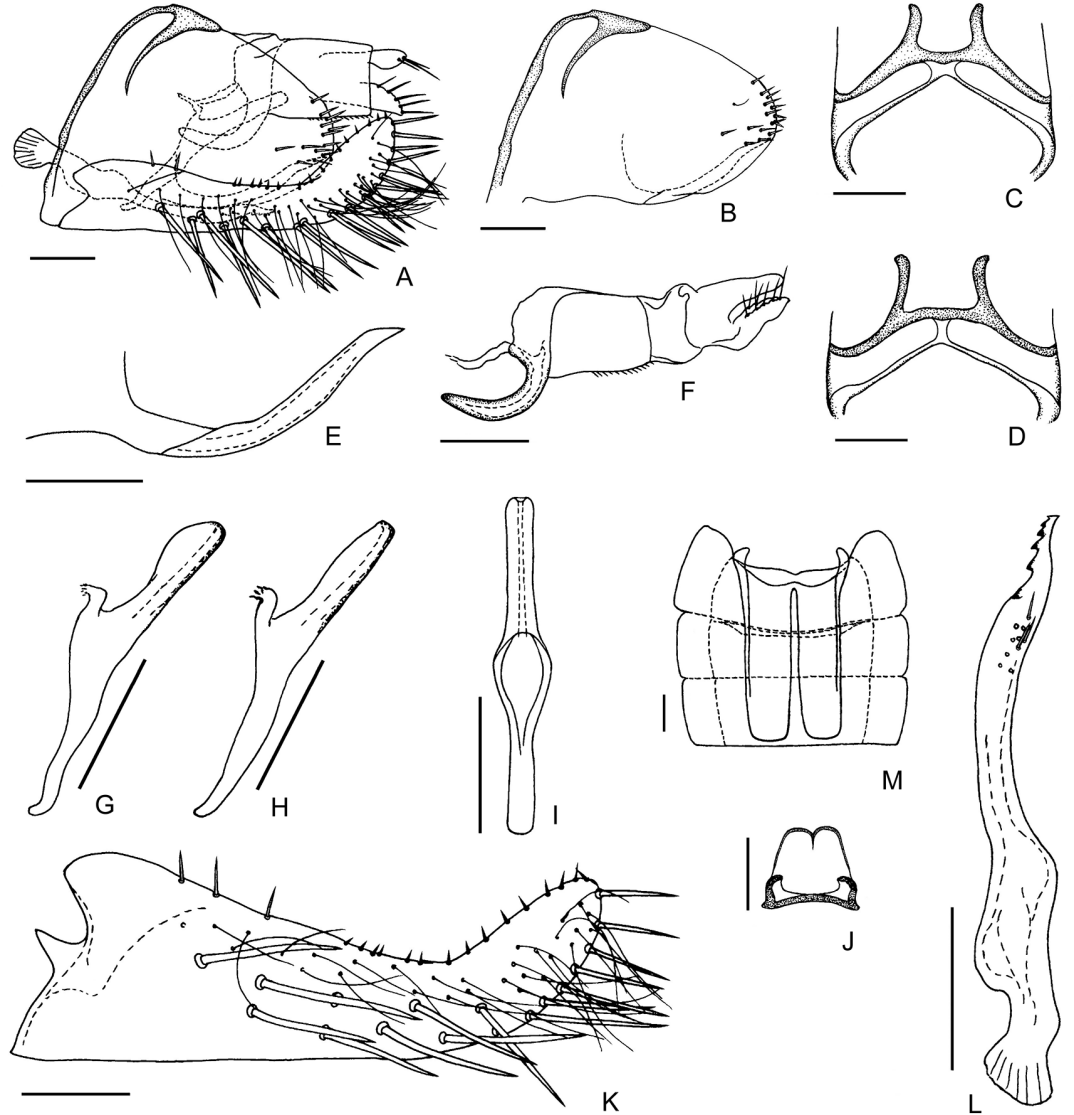
Male genitalia of *E*. (*M*.) *onukii* (specimens from China). (**A**) Male genitalia, left lateral view. (**B**) Male pygofer, left lateral view. (**C, D**) Basal part of male pygofer, dorsal view. (**E**) Ventral pygofer appendage, left lateral view. (**F**) Anal tube and anal styli, left lateral view. (**G, H**) Aedeagus, left lateral view. (**I**) Aedeagus, dorsal view. (**J**) Connective. (**K**) Subgenital plate, ventral view. (**L**) Paramere. (**M**) Abdominal apodemes. Scale bars: (**A)–(M)** = 0.1 mm.

Male basal abdominal apodemes parallel sided, rounded apically, usually surpassing midlength of segment V (Figs 4K and 5M). Pygofer in lateral view with posterior margin rounded, 12–15 stout setae along posterior margin, ventral appendage free from pygofer for most of length, smooth, in lateral view curved upward, apex acuminate and curved posteroventrad, reaching or slightly surpassing posterior margin of pygofer (Figs 4C, 5A, 5B and 5E); in dorsal view sinuate, subapex strongly narrowed and curved laterad, then mediad at apex; dorsum of pygofer with anterior margin sclerotized, bridge narrow to broad, transverse bar and horns well sclerotized, horns slightly curved laterad near apex (Figs 4D, 5C and 5D). Subgenital plates both together broader at base than pygofer in ventral aspect, in profile far exceeding pygofer, base prominently broadened with lamelliform basolateral projection, strongly narrowed distad, apical 1/4 curved dorsad; A-group setae (2–3) near base of plate, shorter than D-group, B-group setae (10–14) small, roughly uniseriate and scattered along dorsal margin in apical half, C-group setae (17–20) starting near 1/4 from base, arranged in double row and merging into single row apically, reaching apex of plate, D-group setae starting basad of C-group macrosetae, roughly biseriate basally with 4 irregular rows distally (Figs 4C, 4D, 4L, 5A and 5K). Paramere in lateral view sinuate, dentifer bowed dorsad, tapered apically, apex truncate, bearing 5–6 distinct teeth and not numerous setae and sensory pits in apical half (Figs 4E, 4F and 5L). Connective lamellate, nearly trapezoidal, slightly longer than maximum width, posterior margin thickened, anterior margin concave medially (Figs 4D, 4F and 5J). Aedeagus in lateral view with preatrium well developed, almost as long as shaft, shaft slender, tubular, straight, diverging from line of preatrium about 30°, with membranous flanges on dorsal side variable in shape, dorsoatrium absent but with pair of ligaments connecting to anal tube laterally, in ventral view aedeagus shaft and preatrium nearly broadened at atrium, gonopore apical (Figs 4C, 4F–4I, 5A and 5G–5I). Anal tube process well developed, in lateral view extended almost half distance to ventral margin of pygofer, smooth, arc-shaped and narrowed distally, apex blunt (Figs 4C, 4J, 5A and 5F).

#### Distribution

China (Shandong, Henan, Gansu, Shaanxi, Xizang, Sichuan, Yunan, Guizhou, Chongqing, Hubei, Hunan, Jiangxi, Anhui, Jiangsu, Zhejiang, Fujian, Guangdong, Guangxi, Hainan, Taiwan, Hongkong); Japan; Vietnam.

### Variation of the Aedeagus

The studied specimens have the aedeagal shaft with variably shaped membranous flanges on the dorsal side both in Chinese and Japanese specimens. These differ from those illustrated for this species by Dworakowska (1971) [32]. Examples are shown in Figs 2H, 2I, 4H and 4I. Such variation occurs in the aedeagal membranes of many *Empoasca* species and probably results from differences in specimen age and preservation.

## Discussion

Tea production originated in southeast China more than three thousand years ago and subsequently became widespread [38]. Tea is now cultivated on large- and small-scale plantations situated between latitudes 41°N and 16°S in more than 34 countries across Asia, Africa, Latin America, and Oceania [39]. Although several scientific names have been applied previously to the main leafhopper pest of tea, this study shows that the tea green leafhopper in China is the same species as in Japan, consistent with the result of Fu *et al*., 2014 (previous molecular work) which suggested that the tea green leafhoppers in Mainland China, Taiwan and Japan are a single species [25]. This finding is consistent with the pathway by which tea culture spread by sea to Japan in the 9th century during the spread of ancient Chinese civilization [40]. However, the broader geographic distribution of this dominant pest remains unclear today, especially in the most important tea planting area in South Asia. For example, in northeast India, the "tea jassid" *Empoasca* (= *Amrasca*) *flavescens* Fabricius has been identified as an important sucking pest in tea plantations [41]. In Viet Nam, *Empoasca onukii*, *Empoasca vitis*, and *Jacobiasca formosana*, have been reported to be present at the same time [42]. Further study is needed to determine whether tea leafhoppers in these countries have been identified correctly. More work is also needed to clarify whether *Empoasca onukii* injures other plants besides tea, or if there are other empoascan species attacking tea shrubs in these areas.

*Empoasca* is by far the most species-rich currently recognized genus of Cicadellidae, with more than 1,000 species names (> 880 apparently valid) in 12 subgenera described so far [3,37]. Nearly 80 species in 5 subgenera are recorded from China [43]. *Empoasca onukii* is now a member of the subgenus *Empoasca* (*Matsumurasca*) which differs from other subgenera in *Empoasca* by the petiolate (stalked) or triangular third apical cell of the forewing, the presence of an angulate lateral projection at the base of the subgenital plate and the aedeagus often (but not always) with paired processes [44]. *Empoasca vitis* (Goëthe), incorrectly identified by Kuoh & Zhang (1988) as the dominant tea pest in Mainland China, is currently placed in the nominotypical subgenus of *Empoasca*, and differs from *E*. *onukii* in several respects (summarized by Qin *et al*. 2014), including characters of the male anal tube appendage, ventral pygofer appendage and subgenital plate [20]. Because of the great difficulty of distinguishing species in *Empoasca* without examining the male genitalia, we strongly suspect that other species of the genus have been misidentified by applied entomologists. Researchers working with *Empoasca* and other leafhopper pests should consult with expert taxonomists in order to ensure that species identifications are accurate.

*Empoasca vitis* is reported as a major insect pest in many European grapevine growing areas. In addition to grape vine, it may be found on deciduous trees in summer and on conifers or evergreens adjacent to vineyards in winter, on which adults hibernate. When temperatures rise, some individuals migrate into vineyards even quite early in spring, with a more continuous migration taking place soon after bud burst of grapevine plants [45]. Chen (1979) speculated that *Empoasca vitis* moved from peach and forest trees into tea gardens in China because of the more extensive and juicy twigs of tea trees [46]. However, true *Empoasca vitis* has never been recorded as a pest of Chinese grapevines and, indeed, the presence of this species in China has still not been confirmed by taxonomists using the recently accepted concept of this species, although Dworakowska confirmed its presence in Japan [36]. As shown here, previous records of *E*. *vitis* in China are probably based on misidentification of other *Empoasca* species. Additional research is needed to determine whether *E*. *vitis* occurs in China on grape vine or other plants.
